# Perceptions around COVID-19 among patients and community members in urban areas in Cameroon: A qualitative perspective

**DOI:** 10.1371/journal.pgph.0001760

**Published:** 2024-02-16

**Authors:** Sylvie Kwedi Nolna, Douglas Mbang Massom, Louis Aristide Tchoteke, Aristide Bille Koffi, Mark Marchant, Palmer Masumbe Netongo

**Affiliations:** 1 Faculty of Medicine and Biomedical Sciences, University of Yaoundé, Yaoundé, Cameroon; 2 Capacity for Leadership Excellence and Research (CLEAR), Yaoundé, Cameroon; 3 Research Department, Youth Action for Health and Social Change, Yaoundé, Cameroon; 4 Faculty of Public Health & Policy, London School of Hygiene & Tropical Medicine, London, United Kingdom; 5 Faculty of Sciences, University of Yaoundé 1, Yaoundé, Cameroon; 6 Molecular Diagnostics Research Group, Biotechnology Centre-University of Yaounde I, Yaoundé, Cameroon; 7 School of Science, Navajo Technical University, Crownpoint, New Mexico, United States of America; Georgetown University, UNITED STATES

## Abstract

At the onset of the COVID-19 pandemic, the Cameroonian government, to abide by international regulations, prescribed preventive measures, which affected many aspects of social, political, economic, and cultural life. However, there needs to be more in-depth exploration of how communities in Cameroon perceived and were impacted by COVID-19. We explored perceptions and misconceptions concerning COVID-19’s impact on urban communities’ daily lives in Cameroon. We conducted semi-structured interviews and focus group discussions with a heterogeneous sample of 25 participants from five different social categories (health personnel, patients with a confirmed COVID-19 infection, close contacts of patients, community members, and community leaders) to assess their perceptions of the disease. Interviews and FGDs were recorded, fully transcribed, coded manually, and analyzed using a thematic analysis iterative coding process. Three main themes were identified: 1) Knowledge of COVID-19: antagonism between disease and invention, 2) Barrier measures imposed by the “dominant culture,” and 3) Impact of COVID-19 on daily lives. Our study revealed perceptions around general knowledge of the COVID-19 pandemic, noting acceptance and observation of government-imposed protective measures while highlighting the significant changes endured in participants’ daily lives. These findings draw attention to the need to develop flexible and appropriate response strategies for different communities. Although Cameroonian populations were not as intensely affected by the burden of the disease of COVID-19 as other regions, they were still compelled to follow static “cookie-cutter" measures that were internationally imposed, affecting their daily lives in ways that seemed disproportionate to their own experiences of the crisis. These findings have potential implications for the legitimacy of public health institutions and responses.

## Introduction

The World Health Organization (WHO) uses an algorithm for deciding whether to declare public health emergencies of international concern (PHEIC), which is defined as "an extraordinary event which is determined to constitute a public health risk to other States through the international spread of disease and to potentially require a coordinated international response" [1]. On March 11, 2020, with more than 170,000 people infected in 146 countries, the WHO announced that the coronavirus outbreak was a PHEIC, joining a list of health emergencies such as H1N1 in 2009, Poliovirus in 2014, Ebola in West Africa in 2014, Zika in 2016 and Ebola in DRC in 2019. Such PHEICs have been described as "an inflection in global health practice" [2]. To abide by International Health Regulations, the head of the government in Cameroon instructed the adoption of preventive measures to curtail the disease. However, by then, the country had only recorded ten confirmed cases. Nevertheless, prevention measures and social distancing were prescribed nationwide. Due to frail health systems, limited resources, and sequelae from previous epidemics and pandemics, at the beginning of the pandemic, African countries were expected to brace for the worst outcomes [3]. These predictions were not realized either in infections or the burden of disease. Ann Kelly describes the "tensions between medical actuality and global health salience" in critiquing the charismatic power of catastrophic J-curve predictions of epidemiological modeling [2]. When the predictions are not borne out, communities sense the gap between "the virus’s mythic countenance" and "the epidemic’s epidemiological realities [2]. During a pandemic, perceptions mold individuals’ knowledge and adoption of safety measures, thus affecting the public’s uptake of government-imposed attempts to control the health emergency. The thrust of early projections of a high burden of disease in Africa amounted to ’evidentiary charisma,’ informing strict top-down response measures that fell out of step with communities’ risk perceptions and lived experiences of COVID-19 in Cameroon and other parts of Africa.

By the end of 2023, the African continent remained one of the geographical zones least affected by this global health emergency. As of November 30, 2023, four years after the beginning of the pandemic, the WHO reports over 772 million cases of COVID-19 worldwide with the African continent claiming 1.2% of them [4]. While the reasons for the apparent low disease burden remain uncertain, early responses by African governments and demographic age structure appears to be the leading factor [3, 5]. The continent claimed only about 2% of all international cases [6]. As of June 26, 2022, Cameroon counted 120,002 cases, leading to 1,932 deaths, and, as of March 16, 2023, the reported mortality rate for Cameroon was 7.17 per 100 000 population [7]. It has been reported that COVID-19 data in sub-Saharan African countries may be underestimated due to flaws in representativeness [8].

Early in 2020, WHO conducted a global fundraising, which led to the development of Strategic Preparedness and Response Plans (SPRP) that support and protect countries such as Cameroon with weak healthcare systems [8]. The SPRP aimed to minimize the impact of the pandemic around the world by interrupting transmission, controlling infection, and facilitating communication to limit disastrous economic and social effects [8]. At the beginning of the pandemic in May 2020, an assessment to evaluate the country’s core capacities to address public health emergencies concluded that the government did not meet International Health Regulations Standards as there was a lack of nationally well-coordinated response efforts to health emergencies [9]. Nevertheless, to abide by international regulations, Cameroonians were obligated to respect government-mandated preventive measures that affected all aspects of social, political, economic, and cultural life despite the low burden of disease in the country. However, as expected, certain perceptions, misconceptions, and misinformation have been wildly spread and embraced in Cameroon, affecting communities’ daily lives. Multitudes of surveys have been conducted by researchers in several African countries quantifying Africans’ awareness, attitudes, and perceptions [10–13], showing the lack of knowledge and misinformation in African communities about the virus, the disease, and the pandemic.

While these quantitative studies have provided an idea of African populations’ awareness of the pandemic, they have also underscored the need to qualitatively understand the impact of the COVID-19 pandemic on the day-to-day activities of these communities. A national six-month online survey conducted in Cameroon found that Cameroonians adhered to government-implemented nationwide measures in the first weeks of the pandemic. Still, this adherence gradually decreased as time passe [14].

Another study conducted in 2020 revealed a high score of knowledge among Cameroonians, but the attitudes and practice scores were lower [15]. From this limited literature, researchers have highlighted a need for additional education surrounding COVID-19, particularly on modes of transmission, control, and prevention [16, 17]. However, exploring how COVID-19 is perceived and experienced by members of the community in Cameroon can better explain the relationship between knowledge and, attitudes and practice. An in-depth exploration of community members’ perception of government-defined health threats provides an understanding of public behavior during the COVID-19 pandemic and provides insight into future health emergencies and other global crises. Considering communities’ knowledge as perhaps in line with, rather than in tension with, attitudes and practices presents an opportunity to learn how response efforts rather than communities might change.

Our study, conducted during the second and third waves of the pandemic (November 2020–June 2021), thus aimed to assess the community’s perceptions of COVID-19 infection in 5 health districts in Douala and Yaoundé cities. Specifically, we appraised perceptions around knowledge of COVID-19 in the community and described individuals’ perceptions and experiences during the pandemic.

This research provides a valuable lens into African communities’ reaction to public health response strategies to pandemics, providing the knowledge necessary to inform further efforts against the COVID-19 pandemic and future global health emergencies.

On May 5, 2023, the WHO Director-General, upon advice offered by the International Health Regulations (IHR) Emergency Committee, determined that COVID-19 was an established and ongoing health issue and no longer constituted a public health emergency of international concern [18].

### Theoretical framework

Herzlich’s theory of social representations of health and illness was used to frame this study. Social representation of health and disease is a concept introduced by Herzlich in 1973 and has been presented in more recent research to understand the relationship between social representations of health and health behaviors. Social representations of health and illness significantly impact individual and collective health behaviors and have been highlighted as an area in which further research is needed [19]. According to Herzlich (1973), social understandings of influence the way individuals perceive and interpret symptoms, seek medical care, adhere to treatment, and engage in health-promoting behaviors [20]. Therefore, understanding these social representations is crucial for promoting health and designing effective health interventions. Herzlich’s theory of social representations of health and illness was selected in alignment with the use of qualitative inquiry to explore the perceptions and knowledge of COVID-19 among individuals in the communities in Cameroon.

## Methods

### Design

In this qualitative study, we used a comprehensive and deductive research design to understand perceptions of COVID-19. Deductive qualitative research involves the “top-down” approach to data analysis by developing of predetermined codes [20]. We selected a deductive approach to support the organization of the data into categories in alignment with the research questions.

We used individual semi-structured interviews and focus group discussions (FGDs), where individual interviews were to explore personal experiences, and FGDs were to examine opinions and beliefs about the subject matter. Interviews and focus groups are the most used data collection methods in qualitative research as they allow for exploring the views, experiences, beliefs, and motivations of study participants [21]. Focus groups allow participants’ experiences to be explored in an interactive setting. To account for both the individual and group dynamics, we collected both interview and focus group data, which were analyzed together to improve the validity and reliability of the results [22].

### Participants

In line with good practice for qualitative studies [23], purposive sampling was used to select study participants. The target was a heterogeneous sample of participants who had been in contact with or had experience with COVID-19, in health centers, households, and the community. This sampling approach allowed for the recruitment of five different social and demographic categories to exploit a wide range of perceptions of the disease at the level of the various strata of society. A total of 25 participants were recruited from five categories: health personnel, patients (with a confirmed COVID-19 infection), contact persons (had been in close contact with a confirmed COVID-19 infected individual), community members, and community leaders (Table 1). All participants took part in the focus groups and were also interviewed. Selected participants were also diversified in terms of their ages and gender. We considered a community leader as a man or woman who was identified by others in the community as someone who is looked up to as a head of an organized group, one who is opinion is widely respected or is known for enabling others to act [24].

**Table 1:** Organization of interviews.

Population	Number
Patients	5
Contacts	5
Health Care Workers	5
Community Members	5
Community Leaders	5

### Data collection

The field visits were preceded by developing a semi-structured interview guide with themes that emerged from previous literature reviews and consultation with a social science subject matter expert. Participants were identified and recruited from private health centers, household, and community visits to participate in the study. A total of 25 participants took part in the study and were interviewed by two research team members: a research assistant and a social scientist. Each interview lasted an average of 30 minutes. Two FGDs were conducted with eight (8) participants in two cities with the highest incidence of COVID-19 in Cameroon (Douala and Yaoundé) for approximately two hours each. The recorded audio files were transcribed in Word format by the research team. A codebook containing a catalog of themes developed from the interviews and FGDs guides was prepared in advance to facilitate coding by referring to some coding manuals [20, 21, 25]. Data coding was performed manually directly on the verbatim in a collaborative manner by the team: The work was organized in a team of 3 three people coding each transcript individually on a shared online document. The coding was then reviewed and harmonized during a workshop with additional research team members (5 people) that revised and finalized the coding.

### Data analysis

After transcription, five independent coders conducted data analysis through several iterative rounds of coding. A consensus of three of five coders was used in establishing the codes and themes. Codes and themes that did not meet this consensus were revised or removed from the final coding scheme. A cycled coding approach was used as suggested by Saldaña for theme development [25]. The cycled coding approach involved seven phases. In the first phase, exploratory coding was conducted independently by each individual coder. This initial data coding was performed through line-by-line manual of the text of the transcripts. Each member of the team involved in the coding highlighted the answers of the interviewee: the theme, the number of the corresponding code, highlighting the relevant quote, and adding one or more observations if necessary. The exploratory coding was intended to identify initial patterns within the data, corresponding with the research question. In the second phase, the results of the independent coding were discussed, supported by memos justifying the initial codes identified by each researcher. The initial codes were discussed and revised until consensus on interpretation was reached. In the third phase, the codes were organized into categories through code grouping. The codes’ organization was based on patterned coding as a cycle of coding in the data analysis process. A coding scheme was then developed based on the grouping and categorization of the codes. In the fourth phase, the categories were reviewed in connection with the research questions, using a focused coding approach. This phase involved revising code groups and supporting quotations from the data collected. After the code groups were developed, the fifth phase involved the development of a codebook based on the established code groups. Descriptions of the themes and subthemes were developed as part of developing the codebook, through discussion among the five coders. Examples of each theme and subtheme were also identified using participants’ quotes extracted from the transcripts. The codebook developed is presented in Table 2 supported by the codebook. The sixth phase involved identifying points of saturation of each theme and subtheme, which was established based on participant consensus of at least 80% for the main themes (*n* = 20), at least 50% for themes (*n* = 13), and at least 30% for subthemes (*n* = 8). In the seventh and final phase, the researchers reflected on the themes and concepts in alignment with the data, research questions, and theory. Based on these reflections, the presentation of results and discussion sections were developed, as presented below.

**Table 2:** Codebook from cycled coding including theme and subtheme saturation.

Main Theme	Theme Description	Participants (*n*)	References (*n*)
Theme			
*Subtheme*			
**A—General knowledge of COVID-19**	Expressed participant understanding of COVID-19	20	128
A1—How the disease showed up		20	63
*A1a—Origin*		7	7
*A1b—Symptoms*		15	30
*A1c—Definition*		15	17
*A1d—Mode of transmission*		5	6
A2—Past experience of the disease		16	64
*A2a—Diagnosis*		15	22
*A2b—Treatment*		13	28
*A2c—Follow up visit*		7	12
**B—Barrier measures**	Actions and behaviors used to protect against COVID	20	97
B1—Individual		20	52
B2—Collective		16	45
*B2a—Social distancing*, *equipment and collective measures*		12	15
*B2b—Lockdown*, *confinement*		5	5
*B2c—Temperature checking*		2	2
*B2d—Handwashing stations*		10	12
**C—Impact of the disease**	Described impacts of COVID-19 on the lives of participants and those in the community	20	198
C1—Economic impact		15	36
*C1a—Decrease of economic activities*		15	26
*C1b—Unemployment*, *technical leave*		5	7
C2—Social impact		20	143
*C2a—Mistrust*		13	27
*C2b—Fear*		18	43
*C2c—Stigmatization*		5	8
*C2d—Behavior modification changes in social norms (hand greetings*, *hugs*, *etc*.*)*		18	53
C2e—School interruption		2	2
C3—Professional changes		10	19
*C3a—Overload of work*, *busy schedules*		7	8
*C3b—Stress*		2	3
*C3c—Decrease of activities*		4	4
*C3d—Increased expenditures*		1	2
C4—Family changes (modification in family activities)		0	0
C4a—Lack of daycare		0	0
C4b—Limited home visits		0	0
**D—Support needed** *	Resources and services needed due to the impact of COVID-19	19	115
D1—Financial and material support		18	42
D1a—Material support in PPE equipment (gels, masks, gloves, etc.)		17	29
D1b—Subsidies and microgrants		7	10
D2—Social support		15	40
D2a—Raising awareness		15	31
D2b—Citizenship (respect of barrier measures, etc.)		5	7
D3—Psychological support		5	14
D3a—Professional counselling		5	7
D3b—Family moral support		3	5
D4—Institutional support		11	18
D4a—Sharing timely and adequate information (from the Government or NGOs)		7	11
D4b—Enforcing imposed restrictions		4	4
**E—Other relevant information** *	Considerations beyond the scope of the research questions highlighted by participants	15	46
E1—Immunization		14	25
E2—Normalization of COVID-19		6	14

*Theme not presented due to lack of saturation

### Rigor

Multiple working sessions were organized to discuss the themes and concepts identified from the rounds of coding. Multiple coders were utilized to support the validity of the identified themes. The team consisted of five researchers, including a social science research expert. As described above, the first level of analysis was performed independently by each researcher during their interviews; then, meetings were held to harmonize the categories and themes to be retained. Results were discussed until consensus on interpretation was reached. Subsequently, the codebook was revised based on the themes found in the first data collected.

### Ethical consideration

Participation in this study was voluntary. Participants were only enrolled after signing a written informed consent form.

Confidentiality was maintained by anonymizing the participant’s identity and data.

This study was approved by the National Ethics Committee of Cameroon (No. 202/07/1265/CE/CNERSH/SP).

## Results

### Theme 1: Beliefs about general knowledge of COVID-19

This first theme highlights how the participants described COVID-19, depending on their experience. Generally, respondents provided a variety of opinions relating to their knowledge of this novel infection, ranging from where the disease originated, a clear definition of the infection and how it can be contracted and its main signs and symptoms, and sharing their doubts concerning whether the virus existed. Patients and their contacts seemed to be well-informed about the origin of the disease, and they had accurate information and knowledge of the COVID-19 infection and its symptoms. However, some of them were still doubtful of the virus’s existence and believed the disease was a made up as a hoax.

“*It’s a virus that appeared already about ten years ago*, *and its current version COVID-19 was first diagnosed in I think November or October 2019 in China*. *But before that*, *there was also SARS-1 which was also other forms of COVID*.”

(Interview Patient contact, Male)

However, some respondents sometimes manifested doubts and tended to minimize the disease.

“*Mentally*, *I perceived this disease as a utopia; it is often said that when misfortune is not next to you*, *you will find it difficult to consider it*. *At first*, *I was informed that COVID exists*, *then I was reassured because although I had been close to the patient*, *I tested negative for the disease*. *So that increased my faith*, *and I still have it*, *because if I give you my personal experience of the flu in question*, *I confess that my body has the capacity to resist the flu*.”

(Focus group patient contact, male).

On the other hand, community leaders and members presented the disease according to information they received in the community or the media. Sometimes, they are mainly influenced by what information is obtained and behaviors they observe in their community.

“*According to the information that we have on radio*, *television and in the newspapers*, *the Coronavirus is a disease that comes from China*. *That’s what they made us understand*.”

(Focus group Community leader, female)

Although healthcare worker participants had no prior training and exposure related to the COVID-19 infection, they had a good understanding of the disease and were knowledgeable about the origin, signs, and symptoms as well as the transmission and prevention methods against the infection.

“*What I know about corona viruses is that they are found in animals*, *and they infect humans… Yes*, *there are many types that have occurred just like the SARS*. *So*, *this one COVID-19 it is just known*, *to be originating from bats and its infecting humans*”

(Focus group Health Care Worker, female)

### Theme 2: Barrier measures of COVID-19

We distinguished between two types of barrier measures: individual protection measures (wearing a mask, washing hands, social distancing); and those imposed on individuals that are more collective measures (curfew, hand washing stations, systematic temperature measurement, limitation of gatherings). Generally, all participants claimed to try to observe the barrier measures, particularly mask-wearing and handwashing. However, some respondents also talked about protective practices in addition to those prescribed by authorities. Concerning the individual protection measures observed by the respondents, health personnel accustomed to wearing a mask as part of their profession quickly complied with the individual protection measures as deemed relevant to protect themselves from the pandemic:

“*What has changed is the wearing of masks that is now universal*. *And it’s true that before*, *in the hospital masks were mandatory in some instances*, *the operating theatre*, *but not everybody*.. *It has changed a lot because everyone wears a mask*.”

(Interview Health Care Worker, female).

Non-health care professional participants also complied with the respect of barrier measures:

“*Personally*, *to protect myself from the coronavirus*, *I always wear a mask*, *whenever I go out and come back home*, *I wash my hands*, *I try to wash my face with soap as well*. *And when I’m in public*, *I try to be able to maintain the barrier measures as prescribed*.”

(Interview Patient contact, male).

“*I avoid people as much as possible*, *I avoid crowds*, *I wear my mask whenever I go out and I respect the one-meter distance; I think that’s what I’ve been told*.”

(Interview Community member, female).

Regarding collective protection measures, participants pointed out protective measures taken either at the level of their workplace, or at the level of public services and public places such as churches, supermarkets, etc.

“*There’s temperature taking*, *which wasn’t done before at the official buildings*, *hand washing points everywhere*. *Yes*, *there are rooms that have been reserved just to put a patient there because it’s reserved for suspected cases of COVID-19*.”

(Focus group Health Care Worker, female).

Participants also mentioned using some protective measures that were not government mandated:

“*I always wear my mask*. *When I come home at night*, *I make my tea with mint crystals which I drink every day before going to sleep and in the morning before going out*. *I buy these mint crystals every three days*.”

(Interview Community leader, male).

### Theme 3: Impact of the disease

Participants described that the COVID-19 pandemic significantly impacted people’s daily life at several levels. This impact is more pronounced on social changes imposed on the community’s daily habits and lifestyle, including restricted social interactions and human relationships. In addition, participants mentioned the impact on their professional lives. The pandemic’s impact on the economy was mentioned regarding the slowdown of economic activities and profitability. For example, health personnel reported lower attendance at health facilities, resulting in reduced revenues. Community members mainly expressed concern about the lack of purchasing power and increased unemployment.

“*The main challenge we faced was the population that reduced in the hospital because of fear and payment difficulties*.”

(Interview Health Care Worker, male)

“*Yes*, *for example*, *prices have increased on the market as you can see*. *Even with a sum of 5*,*000 CFA francs it is not enough to shop and cook for a one-day meal for everyone in the house*”

(Interview Community Member, female)

Sometimes, health personnel reported their experiences in terms of fear of contagion from others, once back home.

“*It’s true that I haven’t lost anyone*, *a family member to the coronavirus yet*, *but when I see others die at work*, *I am also upset*. *Otherwise*, *we are still living in fear that it can kill us*, *it could*…”

(Focus group Health Care Worker, male)

Some Health workers mentioned trust in medicine, considering COVID-19 as a disease like any other that can be cured if measures are taken. However, many others have experienced these various changes from the perspective of strict compliance with a set of measures in the hospital environment.

“*It’s just that we’ve had an increase in personal protective equipment*. *All the staff*, *everyone with their own equipment*, *aprons*, *everything; glasses*, *gloves*. *Everyone had this and it is true that at the beginning*, *the patients had decreased a little but afterwards it returned to normal*”

(Focus group Health Care Worker, male)

### Social and family changes

Many respondents reported that the pandemic had impacted their social relationships and interactions with others and contributed to “cutting the social link” with loved ones (family members, friends, and community members). This perception is widely shared by contact persons, patients, community members, and leaders.

“*Automatically our community is affected because it is not as close as it used to be*. *We no longer have this possibility of being able to deal directly family about certain things*.”

(Interview Patient contact, female)

## Discussion

This study investigated the different perceptions of COVID-19 among five different social categories in the most populous urban areas in Cameroon, Douala and Yaounde. Although the burden of the disease of COVID-19 was not as high in African countries like Cameroon, the findings of our study showed that individuals in Central African communities were as socially affected as any other group around the world. Using Herzlich’s theory of social representations of health and illness as a basis when studying perceptions towards a specific disease, we note that representations were formed from the participant’s experiences but also from information, knowledge, and models of thought that were received and transmitted through tradition and social communication [26]. A previous study conducted during influenza outbreaks found that individuals’ reasoning during a health emergency can be influenced by the perceived effectiveness of health measures, for instance [27]. Our study results showed how participants think about, talk about, and understand the COVID-19 pandemic in terms of social representations.

### Knowledge of COVID-19: Antagonism between real disease and invention

For some of our study participants, the disease was non-existent or exaggerated, the effects of which were amplified by the dominant culture (Governments, local authorities, media, and international community). This perception highlights the suspicions around the disease’s origin, the ambiguities on the treatment, and other conspiracy theories found in the media. Some participants who became ill and were confirmed to be infected with COVID-19 expressed rejection of their illness in the face of what they deemed a trivial sickness. In some instances, our study respondents also found information (found in the media or disseminated by the government) about COVID-19 to be vague and not explicit enough. For some participants, this acquired information was sometimes contradictory to their lived experiences during the pandemic. Several studies have documented the popular perception of SARS-CoV2 emerging from genetic manipulation from a laboratory in China [28, 29]. Our results show communities in Cameroon learning from their lived experiences what the scientific community would take longer to confirm: For multiple still-contested reasons, much of Africa was less susceptible to a high burden of disease from COVID-19 than many other parts of the world.

### Barrier measures imposed by the “dominant culture”

Although our study participants reported observing personal and collective protective measures and to avoid infection, many believed that the facts or information relayed by the media, the national government, and the international health authorities was exaggerated. However, given the strict measures adopted and the expected punishments, our participants felt obligated to respect the barrier measures and the directives imposed by the government, in part due to the *evidentiary charisma* of projections and responsive government decisions. It has even been argued that the COVID-19 pandemic could be perceived as a “Revolution” [14] where the impact of COVID-19 is perceived not only as a disease but a new world order. In other ways, the pandemic can be seen as a social phenomenon that imposes itself on everyone and modifies the way of life on several levels. In a study conducted in the Democratic Republic of Congo, adherence to preventive measures against COVID-19 was found to be suboptimal, despite the compulsory guidelines instituted by the DRC government [13]. Lack of adherence to government-mandated guidelines was blamed on the government’s lack of effort in community engagement. In Cameroon, the dissonance between the official news and the imposition of control measures shows the potential for social learning to undermine government and other power structures when communities’ experiences are in tension with perceived overreactions to disease outbreaks. This incongruity has implications for how scientists inform governments with evidence and how governments use evidence to make decisions in disease response that impinge on communities whose experiences do not align with the original evidence.

### Impact of the disease

According to our study participants, the COVID-19 pandemic has imposed new regulations on social habits and human relationships. Due to its perceived global severity, this health emergency had a profound impact on society, forcing populations all around the world to modify daily habits (social distancing) as well as adopt new ones (confinement), which led to a sort of rupture of social activities and the social life as they knew it. Many of our study participants also reported experiencing a lack of trust in public health institutions which has been corroborated by various studies in other contexts [22, 23, 30]. Therefore, our study participants believed in not having a choice but to learn how to live with this pandemic, integrate the new habits, accept the virus with its multitude of mutations, and accept being vaccinated against it. Initial determination to comply with restrictions and resilience to deal with the economic and social costs gave way to confusion, doubt, and frustration about the intentions of institutions like the government, health research and global health security apparatus whose actions and advice felt out of step with communities’ experiences. When the evidence on the lower burden of disease of COVID-19 in Africa began to change, messaging and restrictions did not change to match, and this expanded the social learning space for conspiracy theories, doubt, and misinformation to explain the gap between the social representation communities were constructing of the threat of COVID-19 in and the one public health officials at multiple levels were communicating. This contrast flags a potential danger of risk communication attempts to exaggerate the threats of a disease to secure compliance with public health measures.

### Decrease in economic activities

Our study participants also noted the decrease in economic activities as a significant negative impact on people’s daily life, as some had lost their jobs or private businesses during this period. Moreover, the impact of this health emergency on African economies has been widely documented. The African Development Bank estimates that economic growth in the continent shrank by 2.1% in 2020 [31]. Also, a previous analysis on the impact of the COVID-19 pandemic on employment in Cameroon found that 60.93% suffered a wage cut while 31.16% faced job suspension, and 7.37% experienced job loss [32].

Although study participants accepted the pandemic regardless of, in some instances, the lack of synchronicity with their personal experience, they admitted to being compelled to endure its impact on their daily lives and their economic activities. Communities’ understandings of the hardship and suffering associated with the economic impact of health restrictions were in tension with the severity of disease that supposedly justified those restrictions. The conceptions of COVID-19 among communities in Cameroon shifted from *a threat that requires action* that inspired compliance and even stewardship of health measures to *a non-serious threat that needs revision* that inspired doubts and undermined the legitimacy of expensive, coercive public health restrictions and the institutions that advocated for them.

### Limitations

The study was carried out in urban areas in two large cities with the highest COVID19 infection rates in Cameroon, and the findings may not apply to rural areas that were less affected. Beliefs and habits in rural communities in Cameroon may be influenced by other exogenous or endogenous factors such as influential members of the community, rites, and ancestral traditions, consumption habits, among others. As interviews and focus group discussions were conducted, recorded, and transcribed in French, the translation process may have obscured the nuances of the original recordings. Despite bilingualism and professional and personal experience with Cameroonian communities, researchers’ overlap in the collection and analysis of the data may introduce some bias. Questions asked during the interviews and focus group discussions may have covered only some aspects of the health crisis.

## Conclusion

Our study highlights in-depth issues around the pandemic as perceived by individuals in various communities in Cameroon. Analysis of semi-structured interviews and focus group discussions with various individuals in Cameroonian cities revealed general knowledge and perceptions of the COVID-19 pandemic noting that acceptance of and compliance with government-imposed protective measures while highlighting the significant changes endured in their daily lives. These findings draw attention to the need to develop appropriate and flexible response strategies for different communities. Cameroonian populations were not intensely affected by the burden of the disease, but as their social representations of COVID-19 were constructed and adapted, they were still compelled to follow static "cookie-cutter" measures that were internationally imposed, affecting their daily lives in ways that seemed disproportionate to their lived experiences of the health crisis. These findings have potential implications for the legitimacy of public health institutions and responses. For other potential areas of research that might shed light on this subject, we suggest an examination of temporary tension between policy or restrictions and community views, and policy makers involvement in efforts to build trust and legitimacy in Cameroon.
